# Efficient mapping of the thalamocortical monosynaptic connectivity in vivo by tangential insertions of high-density electrodes in the cortex

**DOI:** 10.1073/pnas.2313048121

**Published:** 2024-01-19

**Authors:** Jérémie Sibille, Carolin Gehr, Jens Kremkow

**Affiliations:** ^a^Neuroscience Research Center, Charité-Universitätsmedizin Berlin, Berlin 10117, Germany; ^b^Bernstein Center for Computational Neuroscience Berlin, Berlin 10115, Germany; ^c^Institute for Theoretical Biology, Humboldt-Universität zu Berlin, Berlin 10115, Germany; ^d^Einstein Center for Neurosciences Berlin, Berlin 10117, Germany

**Keywords:** connectivity, thalamo-cortical, high-density silicone probe, sensory processing, mouse

## Abstract

The thalamus provides the principal input to the cortex and therefore understanding the mechanisms underlying cortical integration of sensory inputs requires to characterize the thalamocortical connectivity in behaving animals. Here, we propose tangential insertions of high-density electrodes into mouse cortical layer 4 as a method to capture the activity of thalamocortical axons simultaneously with their synaptically connected cortical neurons. This technique can reliably monitor multiple parallel thalamic synaptic inputs to cortical neurons, providing an efficient approach to map thalamocortical connectivity in both awake and anesthetized mice.

## Significance

The thalamus provides the principal input to the cortex and therefore to understand the mechanisms underlying cortical processing, perception and behavior requires unraveling the thalamocortical connectivity in vivo. We here describe an approach for mapping the thalamocortical connectivity in mice in vivo using high-density Neuropixels probes. Tangential insertions of high-density electrodes into the middle layer of the cortex allow detecting action potentials (APs) from thalamic axons simultaneously with somatic APs from postsynaptic cortical neurons. The close physical distance of recorded thalamocortical axons and cortical neurons on the high-density electrode results in a high yield of measured thalamocortical connections in vivo, within single recordings. Thus, this method permits to efficiently map the thalamocortical connectivity in vivo.

The thalamus provides the main afferent input to the cortex and is crucial for its computations (1–8) as well as its functional organization (2, 9, 10). However, how spikes from thalamic neurons are integrated into the ongoing cortical activity in behaving animals remains largely unknown due to technical difficulties in recording synaptically connected thalamocortical neuron pairs in vivo (11). Electrophysiological paired recordings are the gold standard for characterizing monosynaptic connectivity in vivo (1, 3, 7, 12, 13). This technique has revealed important insights into the organizing principles of the thalamocortical pathways (6, 7). However, in vivo paired recordings require a precise alignment between the electrodes in the thalamus and cortex to capture the activity of synaptically connected neuron pairs. This step is technically challenging and therefore limits the numbers of identified connected neuron pairs per experiment (11). Consequently, while important progress has been made over the last decades (2, 3, 14, 15), our overall understanding of the thalamocortical processing in sensory and nonsensory areas remains incomplete and is an active field of research (16).

A method capable of reliably recording the activity of synaptically connected thalamic and cortical neurons in vivo would greatly advance our understanding of thalamocortical processing and could reveal mechanisms underlying perception and behavior. Recording the electrical activity of thalamic neurons where their axons arborize within the cortex, simultaneously with the activity of nearby cortical neurons, would overcome the challenging step of aligning the thalamic and cortical probes in paired recordings and allow to map the thalamocortical connectivity in a more efficient manner. Recently, we showed that high-density electrodes (Neuropixels probes (17) capture the activity of retinal ganglion cell axons within the mouse superior colliculus, allowing a detailed characterization of the retinocollicular synaptic connectivity in vivo (18). Here, we investigated whether high-density electrodes can also record the activity of thalamic axons within the cortex and thus allow to efficiently monitor and functionally map thalamocortical monosynaptic connections.

## Results

### Measuring Thalamocortical Axons within Layer 4 of the Cortex.

**Fig. 1.**
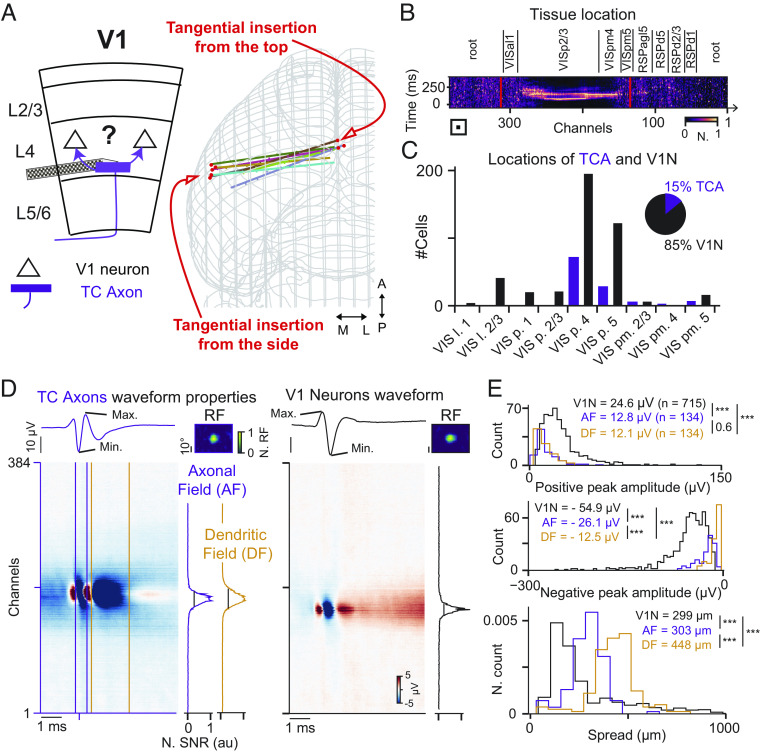
Neuropixels probes capture thalamocortical axons within the cortex in vivo. (*A*) Schematic of thalamocortical afferent (TCA) connecting onto cortical V1 neurons (V1N) (*Left*). Reconstructed trajectories of probe tracks for both insertion angles (*Right*), *n* = 8 insertions, *n* = 8 mice. (*B*) Reconstruction of a single Neuropixels insertion via SHARP-track (*Top*), aligned to the corresponding multiunit activity (MUA, *Bottom*). Abbreviations of nonvisual areas: root: unattributed regions, RSP agl 5: Retrosplenial area lateral granular part layer 5, RSPd 1, 2/3, 5: Retrosplenial area dorsal part layer 1, 2/3, & 5. (*C*) Quantification of the locations of all recorded TCA and V1N within visual cortical layers. Abbreviations of visual areas: VIS l. 1 & 2/3: Lateral visual areal layer 1 & 2/3; VIS p. 1 & 2/3 & 4 & 5: Primary Visual area layer 1 & 2/3 & 4 & 5; VIS pm. 2/3 & 4 & 5: Posteromedial visual area layer 2/3 & 4 & 5. (*D*) Multichannel waveforms (MCW) of a TCA (*Left*) and a V1N (*Right*); TCA properties are quantified on both the axonal field (AF, purple) and the dendritic field (DF, orange); spreads are estimated from the normalized signal-to-noise ratio (SNR) (*Right*). (*E*) Distributions of the positive peak amplitudes (*Top*), the negative peak amplitudes (*Middle*), and the spatial spreads (*Bottom*). ***P = 9.3 × 10^−27^, 2.7 × 10^−31^, 0.641/6.2 × 10^−8^, 5.8 × 10^−24^, 1.4 × 10^−24^/1.7 × 10^−34^, 7.8 × 10^−67^, 1.18 × 10^−22^, *n* = 134 TCAs, *n* = 715 V1Ns, *n* = 8 mice. Comparisons with two-sided Wilcoxon rank-sum test.

**Fig. 2.**
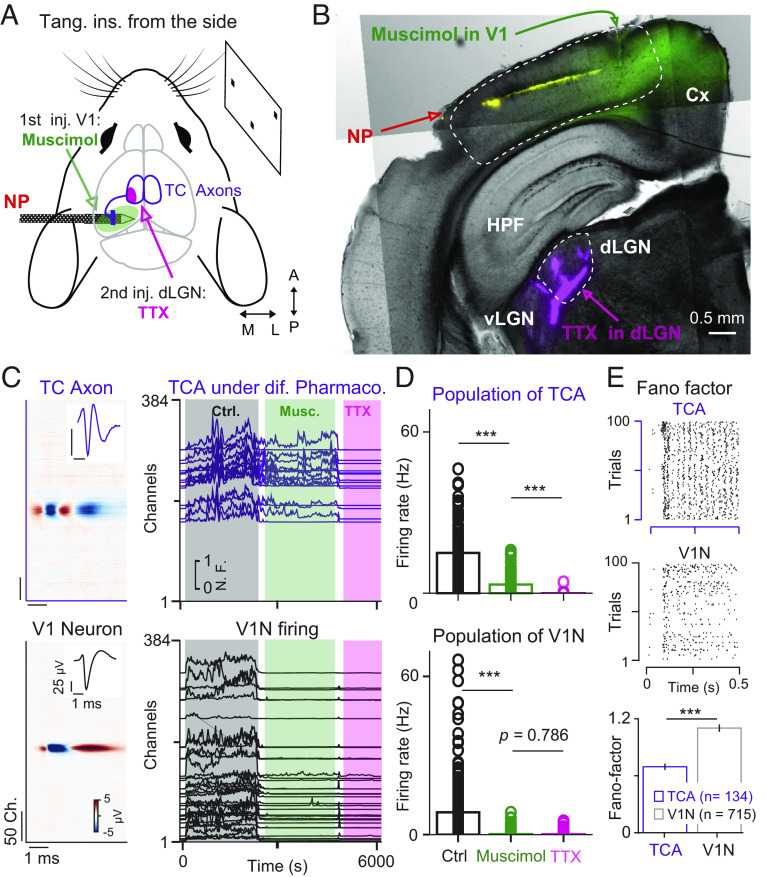
Pharmacology confirms that TCA waveforms originate from dLGN neurons. (*A*) Schematic of pharmacological control experiments. (*B*) Coronal slice showing the Neuropixels probe track (yellow), muscimol injection in V1 (green), and TTX injection in dLGN (magenta). (*C*) Representative TCA (*Top*) and V1N (*Bottom*) with the corresponding MCW (*Left*) and waveform at the peak channel (*Inset*). The activity of TCA and V1N populations is represented over the time course of different pharmacological treatments (*Right*). Each TCA an V1N is shown at its peak channel position (*y* axis): control (gray), muscimol in V1 (green), and TTX in the dLGN (magenta). (*D*) Corresponding firing rates of TCA and V1N during the different pharmacological conditions. ***P = 8.57 × 10^−16^, 9.19 × 10^−16^/ 2.25 × 10^−51^, 0.786, *n* = 86 TCA, *n* = 301 V1N, *n* = 5 mice. Two-sided Wilcoxon signed-rank test. (*E*) Raster plot of TCA (*Top*) and V1N (*Middle*) firing to 100 repeated trials of dense noise stimulation. Comparison of Fano Factor in TCAs and V1Ns. V1Ns show higher variability in firing. ***P = 5.82 × 10^−10^, *n* = 134 TCA, 715 V1N, *n* = 8 mice.Comparisons with two-sided Wilcoxon rank-sum test.

We now report that tangential insertions of Neuropixels probes into layer 4 of the mouse visual cortex (V1) (Fig. 1*A*) can detect extracellularly multipeaked waveforms (Fig. 1*D*) simultaneously with somatic action potentials (APs) of V1 neurons (V1N, Fig. 1 *A*–*D*, see *Materials and Methods* and *SI Appendix*, Extended Data Fig. 1 for optimizing probe placement). The multipeaked waveforms resemble reported signals of thalamocortical axons (19) (TCA), and our results and analysis confirm that they stem from thalamocortical axons within the cortex (see below). The identification of TCA was based on the presence of a second large rebound peak (>1 ms) with a large spread (>10 channels, cf. Figs. 1*D*, 2*D*, and 4*A* and *SI Appendix*, Extended Data Figs. 6 and 7). To characterize the spatiotemporal properties of the TCA waveforms, we distinguished the axonal presynaptic contact field (AF) from its dendritic synaptic contact field (DF), quantifying their amplitudes and spatial spreads on the probe. Both AF and DF have smaller amplitudes compared to the waveforms of V1N (Fig. 1*E*, positive peak amplitude: V1N = 24.6 ± 16 µV, AF = 12.8 ± 9.8 µV, DF = 12.12 ± 6.8 µV, *P* values: V1N to AF *P* = 9.3 × 10^−27^, V1N to DF *P* = 2.7 × 10^−31^, AF to DF *P* = 0.641, negative peak amplitude: V1N = −54.9 ± 35 µV, AF = −26.1 ± 13.9 µV, DF = −12.5 ± 7.9 µV, *P* values: V1N to AF *P* = 6.2 × 10^−8^, V1N to DF *P* = 5.8 × 10^−24^, AF to DF *P* = 1.4 × 10^−24^, two-sided Wilcoxon rank-sum test, *n* = 715 V1N, *n* = 134 TCAs, *n* = 8 mice). In addition, both AF and DF exhibit a wider spatial spread (20) compared to V1N (Fig. 1*E*, spread: V1N = 299 ± 10.5 µm, AF = 303 ± 14.2 µm, DF = 448 ± 17.3 µm, *P* values: V1N to AF *P* = 6.2 × 10^−8^, V1N to DF *P* = 5.8 × 10^−24^, AF to DF *P* = 1.4 × 10^−24^, two-sided Wilcoxon rank-sum test, *n* = 715 V1N, *n* = 134 TCAs, *n* = 8 mice). Overall, the small amplitude of TCA waveforms combined with their larger spread suggests that the high density of recording sites with low noise level (17) is a prerequisite to detect TCA waveforms in the cortex in vivo.

### Multipeaked Waveforms Originate from Thalamocortical Axons.

The spatiotemporal profile of the multipeaked waveforms (Fig. 1 *D*, *Left*) resembles those of thalamic axons making connections to cortical columns. As previously reported by different electric signals recorded in the cortex when averaging on the spike times of simultaneously recorded thalamic neurons: in spikes triggered current sources density (19, 21) and spikes triggered averaged (STA) extracellular waveforms (6, 19, 21, 22). Moreover, the majority of the multipeaked waveforms were recorded in the middle layers of the cortex (Fig. 1*C*), which is the main target layer of thalamic axons. In addition, comparing the trial-by-trial variability during visually evoked activity between V1N and TCA revealed a higher variability in V1N represented by an increased Fano Factor, an established difference between the thalamus and cortex (23) (Fig. 2*E*, Fano Factor: V1N = 1.12 ± 0.04, TCA = 0.71 ± 0.03, *P* = 5.8 × 10^−10^, two-sided Wilcoxon rank-sum test, *n* = 715 V1N, *n* = 134 TCAs, *n* = 8 mice). This circumstantial evidence supports that the multipeaked waveforms originate from thalamic axons. To further test that these waveforms reflect the extracellular field from thalamic axons in the cortex, we performed several causal experiments. This set of experiments shows that 1) TCA waveforms do not stem from local cortical neurons as upon muscimol injections (a GABA_A_ agonist) the TCA waveforms remain active (Fig. 2 *C* and *D*, green, TCA firing rate: control = 16.9 ± 11.4 spikes/s, muscimol = 3.49 ± 4.06 spikes/s, *P* = 8.57 × 10^−16^, two-sided Wilcoxon signed-rank test, *n* = 86 TCA, *n* = 5 mice) while the activity of V1N is almost completely suppressed (Fig. 2 *A*–*D*, green, V1N firing rate: control = 8.7 ± 10.1 spikes/s, muscimol 0.15 ± 0.7 spikes/s, *P* = 2.25 × 10^−51^, two-sided Wilcoxon signed-rank test, *n* = 301 neurons, *n* = 5 mice). Note that while TCA remain active, their firing rate is reduced upon muscimol injection, which could be due to the reduction of corticothalamic feedback (24, 25) or the activation of presynaptic GABA_A_ receptors in thalamic axons (26) as we injected large amounts of muscimol (*Materials and Methods*). 2) TCA waveforms originate from the dorsal lateral geniculate nucleus (dLGN), as their activity is abolished upon injection of Tetrodotoxin (TTX, a blocker of sodium voltage-gated channels) in the dLGN (Fig. 2 *A*–*D*, magenta, TCA firing rate: muscimol = 3.49 ± 4.06 spikes/s, TTX = 0.019 ± 0.06 spikes/s, *P* = 9.19 × 10^−16^, two-sided Wilcoxon signed-rank test, *n* = 86 TCAs, *n* = 5 mice). 3) To further support that the multipeaked waveforms originate from thalamic axons, we performed experiments to electrically stimulate neurons directly in the dLGN (19, 21, 22) using a tungsten wire that was stereotactically placed within the ipsilateral dLGN while extracellularly recording TCA waveforms in the cortex (*SI Appendix*, Extended Data Fig. 6*A*). Multiunit activity (MUA) in the visual cortex exhibits clear responses upon electrical stimulation with a delay from 1 to 5 ms (*SI Appendix*, Extended Data Fig. 6*B*). TCA and V1N respond both to the electric stimulation (*SI Appendix*, Extended Data Fig. 6 *D*–*F*) with a shorter response onset in TCAs (*SI Appendix*, Extended Data Fig. 6 *D* and *E*, average first spike 2.52 ± 0.17 ms, *n* = 19 TCA, vs. 5.06 ± 0.28, *n* = 25, *n* = 1 mouse). In summary, the responses of multipeaked waveforms to the electrical stimulation in dLGN strengthen our interpretation by a more classical approach (19, 21) which supports the conclusion that these multipeaked waveforms are TCA waveforms originating from neurons in the dLGN.

### Comparing Thalamocortical and Corticocortical Axonal Electrical Signals.

**Fig. 3.**
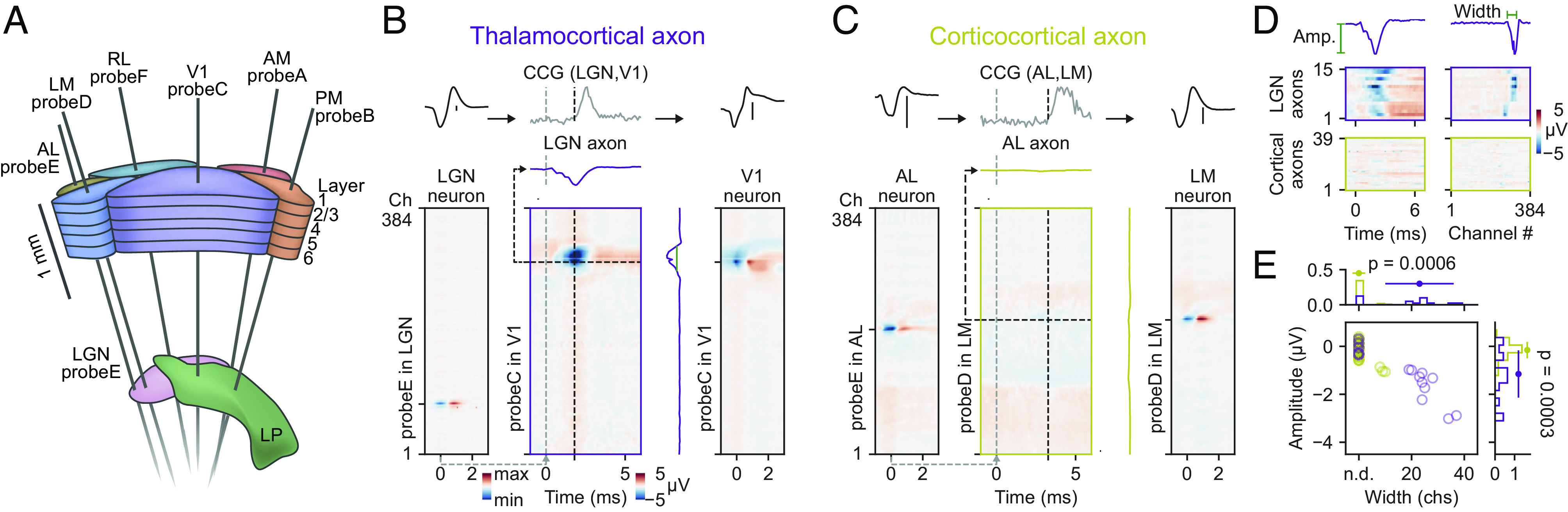
Comparing the STA waveforms evoked by long-range thalamocortical and cortico-cortical axons. (*A*) Schematic of the Neuropixels recording trajectories targeting cortical and subcortical visual circuits in the AI dataset. Adapted from (27) (*B*) dLGN evoked axonal STA waveforms in V1. The dLGN neuron (*Left*) is monosynaptically connected to a V1 neuron (*Right*), identified by a peak in the CCG (*Middle*). Note the pronounced axonal evoked STA waveforms at the location of the postsynaptic V1 neuron on the probe in V1 (*Middle*). (*C*) STA waveform of a long-range cortico-cortical axon, connecting a neuron in visual cortex area AM with a neuron in visual cortex area LM. Note the absence of any axonal evoked signal at the location of the postsynaptic cortical neuron. (*D*) Axonal evoked STA waveforms across time and space for all dLGN and cortical axons studied here. dLGN axons show a negative peak in space and time (*Top*) while the evoked STA waveforms of cortico-cortical axons are weak or absent (*Bottom*). (*E*) Amplitude vs. width of the evoked STA waveforms of thalamocortical (violet) and cortico-cortical (yellow) axons. The schematic is adapted from ref. 27. *P* = 0.00056 for the amplitude, *P* = 0.00029 for the width, *n* = 15 dLGN to cortex pairs, *n* = 7 sessions/animals, *n* = 39 corticocortical pairs, *n* = 6 sessions/animals, two-sided Wilcoxon rank-sum test.

These causal experiments altogether confirm that multipeaked waveforms stem from axons that can originate from the dLGN. However, these experiments do not disprove that multipeaked waveforms could be from local or long-range corticortical axons. To address this critical question, we directly compared the spike-triggered averaged waveforms (6, 19, 21, 22) evoked by thalamocortical vs. long-range cortico-cortical axons. To do so, we took advantage of the publicly available large-scale Neuropixels dataset from the Allen Institute (AI) (*Materials and Methods*). In this dataset, up to six Neuropixels probes were inserted into the mouse brain in each experimental session to record simultaneously the activity from hundreds of neurons in multiple visual cortical regions (AL, LM, RL, V1, AM, and PM) as well as neurons in subcortical visual nuclei (LGN and LP) (Fig. 3*A*). This dataset thus offers the opportunity to measure and compare STA waveforms (6, 19, 21, 22) (*Materials and Methods*) evoked by thalamocortical and long-range cortico-cortical axons. To identify long-range connections within this dataset, we calculated the spike-train cross-correlogram (CCG) between pairs of neurons that were located in different brain regions, e.g., dLGN and V1. Putative monosynaptic connections were detected by a transient peak in the CCG (1, 5–7, 11, 18) (Fig. 3 *B*, *Top* and *Materials and Methods*). We repeated this step for a large number of experiments and neuronal pairs resulting in a set of thalamocortical and long-range cortico-cortical connections (*n* = 56 experimental sessions with neurons recorded in multiple cortical regions, 3,292,834 tested connections, *n* = 155 detected cortico-cortical connections; *n* = 30 sessions with simultaneously recorded neurons in the dLGN and V1, 82,588 tested connections between dLGN and V1, *n* = 23 detected dLGN-V1 connections). Because the presynaptic and postsynaptic neurons were located in separate brain regions, their somatic AP was recorded on different Neuropixels probes, e.g., the dLGN neuron shown in Fig. 3*B* was recorded on probeE (Fig. 3 *B*, *Left*) while the V1 neuron was recorded on probeC (Fig. 3 *B*, *Right*). To capture the axonal evoked STA waveforms on the Neuropixels probe of the postsynaptic neuron, we randomly selected 20,000 presynaptic spike times and averaged the spike band signal of the postsynaptic Neuropixels probe at these spike times (Fig. 3 *B*, *Center*). This analysis showed that dLGN axons evoke a negative waveform at the location of their synaptic contacts within the cortex (Fig. 3 *B*, *Middle*), reproducing previous findings (19, 21, 22). In contrast, the STA waveforms of long-range cortico-cortical axons at the location of their synaptic contacts are very weak or even absent (Fig. 3*C*). To quantify these observations, we measured the amplitude and spatial spread of both STA waveforms (Fig. 3 *D*/*E* and *Materials and Methods*). The peak lag in the CCG was used as a guide to identify the time of the expected evoked STA waveforms (time of measurement: peak lag – 1 ms). Moreover, the location (peak channel) of the postsynaptic cortical neuron provided the spatial position on the probe to measure the axonal evoked STA waveforms over time (*Materials and Methods*). This analysis shows that thalamocortical and cortico-cortical axons evoke distinct STA waveforms at the site of their postsynaptic contact: Thalamocortical axons evoke measurable STA waveforms on the Neuropixels probes (amplitude = −1.114 ± −0.984 µV, *n* *=* 15 TCA, *n* = 7 sessions) covering multiple recording sites (width = 23.00 ± 12.947 recording sites). In contrast, the evoked STA waveforms by long-range cortico-cortical axons were weak or even undetectable (amplitude = −0.047 ± −0.341 µV, *n* = 39 CCA, *n* = 6 sessions) and the spatial spread on the probe consequently small (width = 0.0 ± 2.409 recording sites) (Fig. 3 *D* and *E*). These findings indicate that the electrical footprints of thalamocortical and long-range cortico-cortical axons are distinct (amplitude: *P* = 0.0005663772, width: *P* = 0.0002934278, see *SI Appendix*, Extended Data Fig. 4 for more examples).

Finally, we investigated whether corticocortical axonal signals can be detected from local cortico-cortical connections. To that end, we identified connected pairs within V1 in our own dataset using CCG analysis (*n* = 267 detected connected pairs). To assess whether the postsynaptic evoked spikes were preceded by an axonal waveform, we extracted only the spikes in each V1N firing which match their monosynaptic delay (*SI Appendix*, Extended Data Fig. 5). This analysis revealed the presence of the waveform of the other connected neurons but failed to reveal any further waveforms, suggesting that also within the local cortical circuit cortico-cortical axons have an evoked electrical signal below the measurable limit of our recording method. In summary, the difference in the evoked STA waveforms of thalamocortical and cortico-cortical axons further supports that the multipeaked waveforms, that we measured in the middle layers of the cortex using tangentially inserted Neuropixels probes (Figs. 1 and 2), are of thalamic origin.

### Efficient Mapping of Thalamocortical Monosynaptic Connections.

**Fig. 4.**
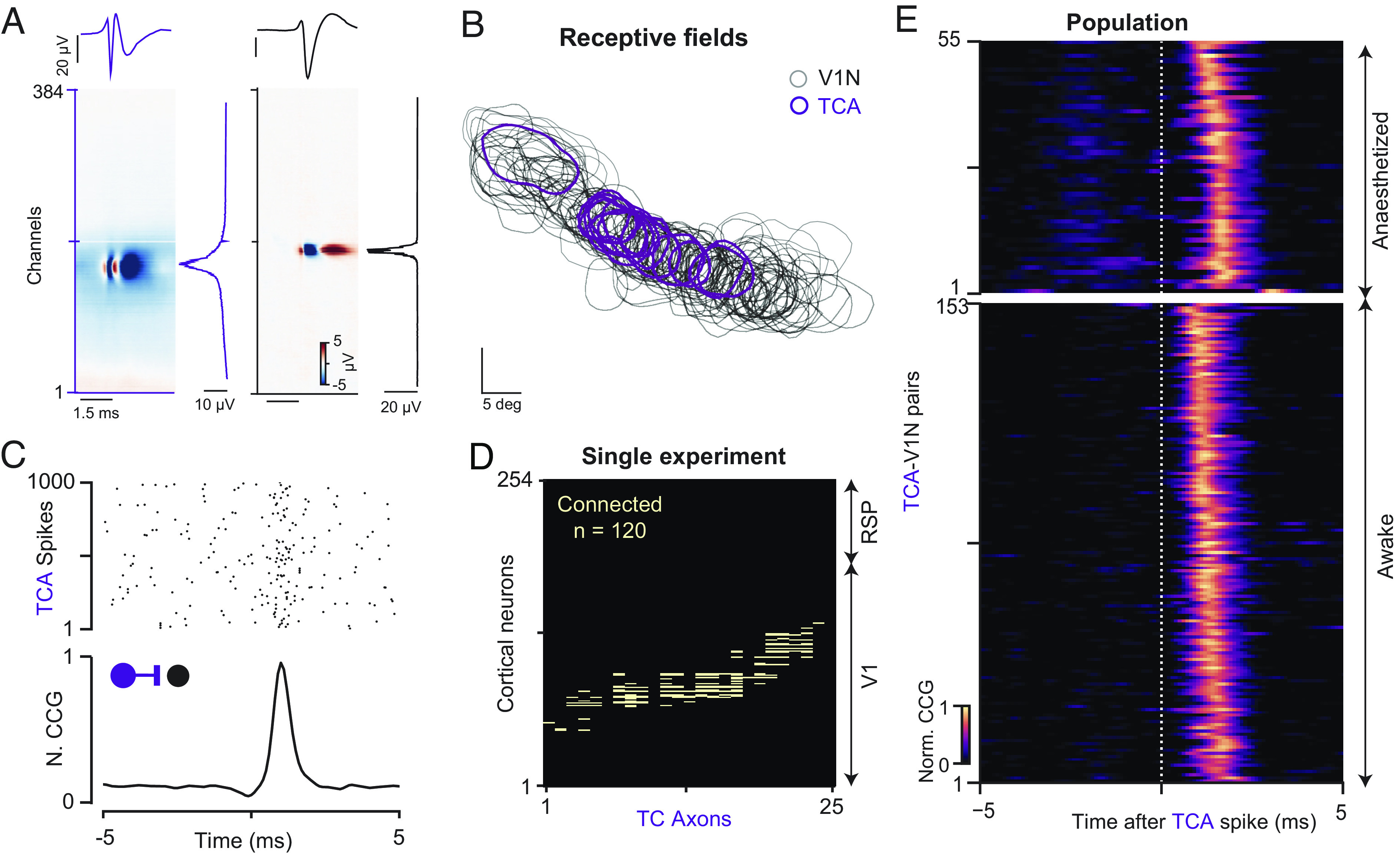
Efficient mapping of thalamocortical monosynaptic connectivity in anesthetized and awake mice. (*A*) Multichannel waveforms (MCW) of a TCA (*Left*) and a neighboring V1N (*Right*) with their respective amplitude profiles. (*B*) Overlapping receptive fields of a population of TCA and neighboring V1N. (*C*) Raster plot showing V1N responses of a connected TCA-V1N pair triggered on 1,000 independent TCA spikes (*Top*) and the corresponding CCG (*Bottom*). (*D*) Connectivity matrix from a single awake recording containing 120 simultaneously detected monosynaptic contacts between 25 TCAs and 254 V1Ns. (*E*) CCG profiles of total measured monosynaptic TCA-V1N connections in anesthetized (*n* = 56 connections; *n* = 6 mice) and awake mice (*n* = 153 connections; *n* = 2 mice).

**Fig. 5.**
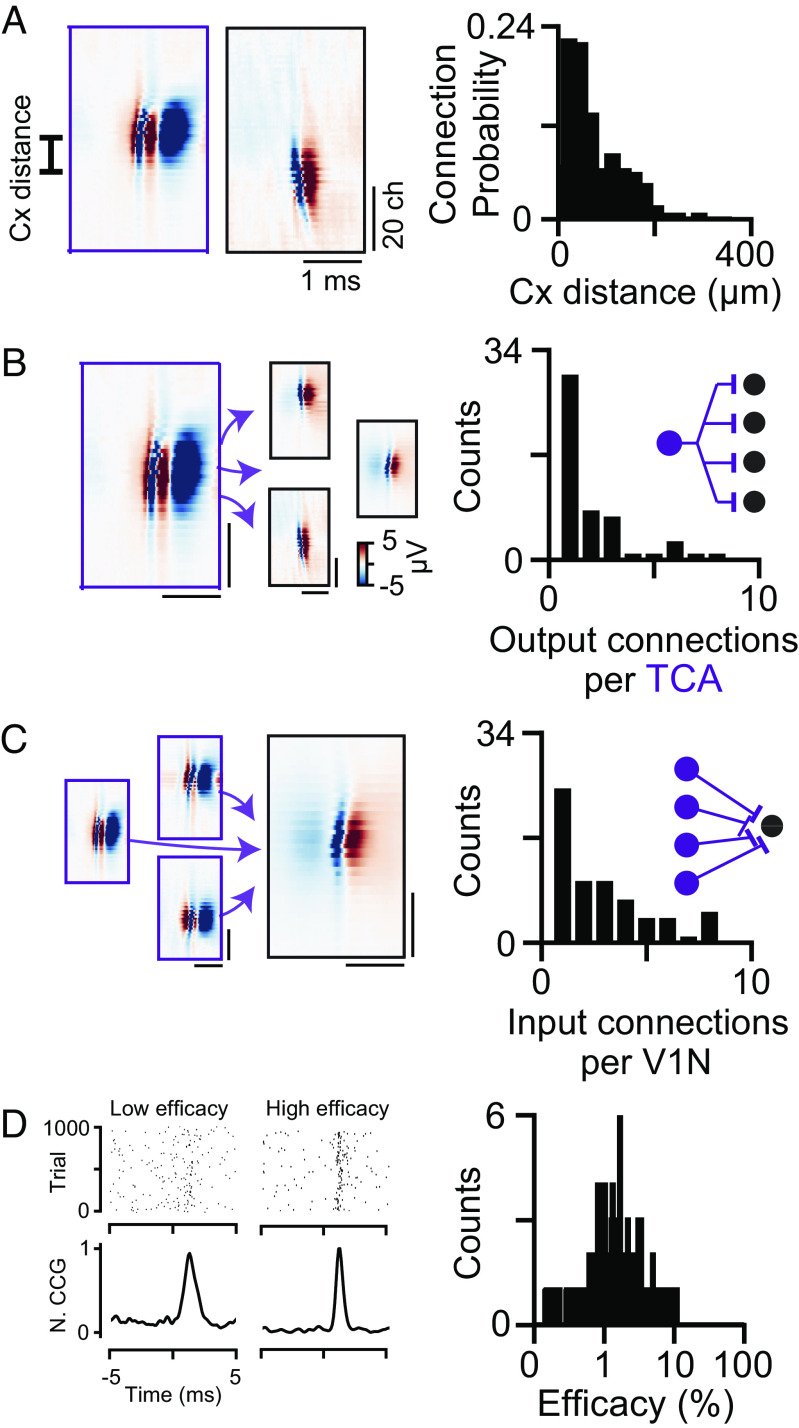
Properties of thalamocortical monosynaptic connectivity. (*A*) MCW of connected TCA and a V1N (*Left*). Distribution of peak channel distances between the connected TCA and V1N (*Right*). (*B*) Distributions of diverging TCA and (*C*) converging V1N (*Right*) connections. (*D*) Examples of weak and strong connections, (raster plot and normalized CCG) and distribution of connection efficacy (*Right*).

Recording the activity of thalamic axons and V1 neurons simultaneously using two electrodes permits to detect connected thalamocortical pairs (Fig. 3); however, the yield of identified connections is usually very low (11). For example, across the 30 sessions in the AI dataset with simultaneously recorded dLGN and V1 neurons, we could only detect 23 dLGN-V1 pairs (23 detected connections in 82,588 tested connections, probability of finding a dLGN-V1 connection = 0.0278%, *n* = 30 recording sessions). In contrast, by employing the tangential recording approach to measure thalamic axons directly within the visual cortex permitted to detect a larger number of monosynaptically connected pairs (Fig. 4, 205 detected connections in 15,801 tested connections, probability of finding a dLGN-V1 connection = 1.28%, *n* = 8 mice), even within individual recordings (Fig. 4 *A*–*D*). As expected, connected TCA-V1N pairs are within close proximity in the cortical tissue (Fig. 5*A*, median distance within the cortex = 62.1 µm, first quartile = 40 µm, third quartile = 121 µm, *n* = 208 connected pairs, *n* = 8 mice). The high yield of identified connections allows to capture divergent (mean identified diverging contacts between single TCA and V1N = 3.41 ± 0.05 connections) and convergent connections (mean identified converging contacts between TCA and single V1N = 3.1 ± 0.03 connections) (Fig. 5 *B* and *C*). Finally, we estimated the spike transmission efficacy (1, 13, 18) as an in vivo measure of the thalamocortical connection strength (Fig. 5*D*, median efficacy = 1.8%, first quartile = 1.2%, third quartile = 3.1%, *n* = 208 connected pairs, *n* = 8 mice), confirming previous reports that individual thalamic neurons have a low probability in driving cortical neurons to spike (1, 3, 6, 7, 19, 21, 22).

## Discussion

In our previous work, we have shown that high-density electrodes extracellularly capture the electrical activity of retinal ganglion cell axons in the midbrain of mice and zebra finches (18). In this study, we now confirm the feasibility of extracellular detection of afferent axons within the cortex of head-fixed awake and anesthetized mice. Several lines of evidence support this conclusion: 1) The characteristic multipeaked waveform of the axons resembles that of thalamocortical axons (19, 21, 22) (Fig. 3*B*). Moreover, the spatial spread of these waveforms within the cortex (Fig. 1) is larger compared to the waveforms of the RGC axons within the midbrain (18), in line with the anatomy of dLGN axons (20) and RGC axons (28). 2) The multipeaked waveforms activity remained after inhibiting local cortical neuronal activity with muscimol. 3) The multipeaked waveforms activity was abolished after injecting TTX in the dLGN (Fig. 2). 4) Furthermore, in a separate set of experiments, electrical stimulation within the dLGN evoked multipeaked waveforms before V1N (*SI Appendix*, Extended Data Fig. 6). 5) Finally, in contrast to the negative STA waveform evoked by thalamocortical axons, the STA waveform evoked by long-range cortico-cortical axons is small or even absent (Fig. 3 *B*–*E*).

Modeling work has shown that the axonal morphology plays a key role in generating extracellular signals (29) supporting the observation that thalamocortical axons produce a strong electrophysiological signal likely due to their denser arborization within layer 4. In contrast, the morphology of different cortico-cortical axons is more distributed spatially (30) providing a possible explanation of why these axons evoked only weak signals (Fig. 3). Future studies using either a combination of patch-clamp and Neuropixels insertions could provide the missing evidence to answer why cortico-cortical axons evoke weak signals.

The submillisecond temporal resolution of Neuropixels probes allows for the temporally precise tracking of axonal and neuronal activity simultaneously. Consequently, the CCGs between these axons and neurons can reveal monosynaptic connections (Fig. 4). The dense sampling of thalamic axons and cortical neurons by a single high-density electrode directly in cortical layer 4 resolves the challenging alignment step and thereby allows to efficiently map the thalamocortical connectivity on a large scale in vivo (mean number of connections per recordings: awake mice = 76.5 ± 30.7 connections, *n* = 2 mice; anesthetized mice = 9.3 ± 3.5 connections, *n* = 6 mice). In addition, the high spatial resolution of Neuropixels probes permits to synchronously monitor other neighboring axons within the cortical tissue (Fig. 5 *B*–*D*): reaching up to 120 functional monosynaptic thalamocortical synapses between 25 TCA and 254 V1N within a single recording (Fig. 4*D*). These numerous identified monosynaptic contacts are detected within close physical distance between the axon and the connected V1N, producing both multiple diverging and converging contacts (Fig. 5).

In summary, we demonstrated that tangential insertions of high-density electrodes into cortical layer 4 are a reliable and efficient method for mapping and characterizing monosynaptic thalamocortical connections in vivo. Thus, this approach is a promising technique for studying numerous thalamic inputs to cortical neurons during potentially a wide range of rodent behaviors.

## Materials and Methods

### Animals, Surgery, Insertion, and Staining.

Agreements from the local authorities were obtained for the anesthetized (*n* = 7) and the awake head-fixed (*n* = 2) experiments (Landesamt für Gesundheit und Soziales Berlin–G 0142/18). Maximum attention was focused on minimizing the number of used animals. Adult C57BL/6J mice were obtained from a local breeding facility (Charité-Forschungseinrichtung für Experimentelle Medizin). For the two awake mice, a headpost was implanted ~1.5 wk prior to the recording under isoflurane anesthesia, painkiller was provided for 3 d after implantation, then mice were habituated with daily sessions gradually increasing duration on the setup from 30 s to 2 h, then adding exposure to classical visual stimuli, i.e., sparse noise, moving bars, moving gratings and full-field chirp stimulus.

On the recording day, the mouse was induced with a controlled level of isoflurane (2.5% in oxygen Cp-Pharma G227L19A). The mouse underwent surgery in a stereotactic frame (Narishige) where the isoflurane level was gradually lowered (0.7 to 1.5%) with constant monitoring of the absence of both responses to tactile stimulations and vibrissa twitching. During surgery, the temperature was constantly monitored (FHC-DC), the eyes were covered with ointment (Vidisic), meanwhile a headpost was positioned, and a dental cement-based crown (Paladur, Kuzler) was built around the skull including a grounding wire. During recording, the crone was filled with a maximal amount of PBS (phosphate buffer saline solution) to improve grounding and reduce electrical noise. For the insertion from the side, regular renewal of PBS during recording may be required. For the tangential insertion from the side, enough room was prepared on the lateral side of the crown for the insertion to enter the brain below the lateral cranial ridge. Once the headpost was solidified, and all craniotomies were finished for the desired experiment(s), the mouse was transferred into the setup to proceed with the recording. For the awake recordings, the mouse recovered from craniotomy surgery for 1 to 2 h before proceeding to the experimental setup.

Two different insertions are proposed to reliably achieve thalamocortical axons measurements in the visual cortex: a) the insertions from the top (Figs. 1*A* and 2 *A*–*E*)—advised for awake recordings—with a negative 15 to 20° from the azimuthal plane (within a coronal plane) or b) lateral insertions (Fig. 1*A*) with a positive 15 to 20° from the azimuthal plane (within a coronal plane). Awake animals often groom their head during the experiment; therefore, tangential insertion from the top will avoid the animal grasping the probe and breaking it. For anesthetized recordings, the more invasive lateral insertions can then be performed. To avoid bending the probe upon tissue contact, the grounding solution (PBS) was temporarily removed. All stereotactic coordinates are reported from lambda, in the anteroposterior (AP), the mediolateral (ML), and the dorsoventral (DV) axis. Insertions from the top enter the brain at 0 to 900 μm AP, +250 to 0 μm ML, and −100 to −500 μm DV, while insertions from the side enter the brain at 0 to 900 μm AP, −5,000 to −4,000 μm ML, and −2,000 to −500 μm DV from lambda. In both implantations, the probe was inserted for at least 3,000 to 4,000 µm and then withdrawn by 50 µm to release mechanical pressure. After the probe settled in the tissue for ~10 to 20 min, a short sparse noise stimulus was displayed to map the visual receptive fields (RF). This first short recording was also used to estimate the placement of the tangential insertion within the cortical layer 4 (cf. below semionline detection of thalamocortical axons). If thalamocortical axonal waveforms were found in visually driven channels (*SI Appendix*, Extended data Fig. 1) the data acquisition was started. A set of visual stimuli was presented, followed either by the different pharmacological applications (cf. below) or, in case of the awake mice experiments, the visual stimuli was repeated. Once the recording was stopped, the Neuropixels probe was partially removed, coated with DiI (Abcam-ab145311, in EtOH), and reinserted to the same depth to stain the recording track in the tissue. Mice were then killed with an excess of isoflurane (>4%) followed by a cardiac perfusion with PBS solution and 4% paraformaldehyde (PFA). Brains were left in PFA overnight and stored in PBS until slicing (Leica VT1200 S) and mounting (100-µm slices, DAPI-Fluoromount-G Biozol Cat. 0100-20). Mounted brain sections were further analyzed to perform the 3D reconstruction of the probe’s location in the tissue using SHARP-track (29) and to relate our staining to the Allen Mouse Brain Common Coordinate Framework. We used the entry and exit point of visually driven activity from the MUA as functional markers of the outer boundaries of the visual cortex (Fig. 1*B* in red) to align and scale to the cortical layers and regions identified by SHARP-track. Consequently, each channel on the probe was assigned to a tissue location to determine the location for each V1N and TCA (Fig. 1*C*).

### Pharmacological Applications.

In the majority of the dataset (5 out of 9 recordings), pharmacological solutions were applied to confirm the origin of the measured TCA waveforms in V1. A first set of visual stimuli set was presented before lowering the injector (Drumond, Nanoject II) vertically right next to the probe at 500 to 1,000 µm ML, 400 to 0 μm AP, −1,100 to −1,400 μm DV from lambda, to inject 100 nL of muscimol solution (Abcam, ab120094, 2.5 mM in PBS) containing a dye (Cholera Toxin subunit B, Alexa 488 Conjugate -C22841, Invitrogen). Upon muscimol injection and diffusion (~5 min), a clear reduction of ongoing spontaneous cortical activity should be observed. In case of insufficient reduction of cortical neuronal activity, a second injection, either in the same location or in another direct neighborhood of the Neuropixels probe, was applied. Once sufficient reduction of cortical activity was obtained the visual stimulus set was presented again followed by a second pharmacological injection of TTX (100 nL, Biozol-HB1034, 100 μM in PBS) with the same dye in the dLGN at 2,300 µm ML, 1,700 μm AP, −4,000 μm DV from lambda. Finally, a chirp stimulus was presented after injection of TTX to confirm the loss of visually driven activity in V1. As the same staining was used in both injections, pseudocolors were arbitrarily attributed in the different brain regions for easier illustrations (Fig. 1*G*). Once the data have been preprocessed, we selected the channels on the probes exhibiting both visually driven activity and pharmacological modulations, all clusters outside of this area of interest were excluded from further analysis.

### Electrical Stimulations.

In a subset of experiment (*n* = 3, 1 successful), electrical stimulation was used to evoke TCA activity and monitor the delay between the TCA and V1N. First, a tungsten wire was glued inside a glass pipette and placed within the dLGN at 2,300 to 2,700 µm ML, 1,400 to 1,700 μm AP, and −3,000 to −3,500 μm DV from lambda. Single biphasic short (20 μs) electrical pulses with increasing intensity were applied until the pulses produced reliable responses in V1 (Isolated Pulse Stimulator from A-M System, model 2100, 20 μs biphasic pulses of 1 mA intensity. 250 mΩ tungsten wire). Relocation of the electrical wire was sometimes required to maximize responses in V1. Once the wire was in the correct position, regular stimulation at intervals of 20 s was applied for the desired numbers of repeats. The unperfect alignment of the electrical stimulation glass pipette to the target position area can lead to electrically driven activity elsewhere in the cortex. Post hoc analysis should combine in the same recorded regions electrical driven activity together with TCA waveforms, which dismissed two recordings (2/3). The electrical stimulation caused an artifact on the Neuropixels probe illustrated in the MUA activity (*SI Appendix*, Extended Data Fig. 6*C*). Consequently, spiking activity that occurs during the artifact period (−0.25 to +1 ms for each stimulus) was removed from further analysis (*SI Appendix*, Extended Data Fig. 6 *E* and *F*). As indicated in red, TCA and V1N within the two red lines (in red, *SI Appendix*, Extended Data Fig. 6*B*) were kept for further analysis (*SI Appendix*, Extended Data Fig. 6*B*). Furthermore, to avoid confusion of delays between first and second or third spikes sometimes elicited by the electric stimulation, we limited the measures of delay to the very first spike observed during an electric stimulation period (in blue, *SI Appendix*, Extended Data Fig. 6*F*).

### Hardware, Software, and Visual Stimulation.

Neuropixels probes (version 1.0) signal was acquired on a PXIe system (National Instrument NI-PXIe-1071) using Open Ephys acquisitions software (www.open-ephys.org). The raw signal was split and stored in the local field potential (0 to 300 Hz) and the AP band (300 to 3 kHz). Ecephys was used to calculate standard cluster quality metrics (*SI Appendix*, Extended Data Fig. 3, https://github.com/AllenInstitute/ecephys_spike_sorting). Except for sorting with Kilosort (31) (https://github.com/MouseLand/Kilosort) which was done in MATLAB 2018 and 2019 (www.mathworks.com), all data analysis was performed in Python 3 (www.anaconda.com); statistical tests were performed using either the two-sided Wilcoxon rank-sum test except for the pharmacological recording where we use a two-sided Wilcoxon signed-rank test (Fig. 1*I*). *P* values are indicated as follows: “***” indicates a *P* value below 0.001. The visual stimuli were displayed on a monitor screen (Dell, calibrated at refresh rate = 120 Hz, mean luminance = 120 cd/m^2^). The visual stimulus set contained the following stimuli: two sparse noise stimuli with either dark or light targets (15° size, 2 targets/frames of 100 ms, 20 repeats for each position on a grid of 36 × 22 squares), moving bars (white bars on black background, 10° in width, 12 directions, fixed speed of 90 deg/s), followed by the final full-field chirp stimulus (18). For each exposed image, a TTL signal was sent from the stimulus computer to the PXI system and recorded via the PXI-8381 module simultaneously with the Neuropixels data to allow alignment of the visual stimuli to the spike times.

### Semionline Detection of Thalamocortical Axons.

To ensure the tangential insertion of the Neuropixels probe through the cortical layers 4, and the quantity of thalamocortical axons in the recording, we advise to estimate the potential number of visually driven TCAs. To do so, the short sparse noise stimulus was displayed, and neuronal activity was recorded (32). The first main verification is to use an adaptation of the “make_fig.m” script from Kilosort (33) (*SI Appendix*, Extended Data Fig. 1, compatible with KS 2 & 2.5) (https://github.com/KremkowLab/Cortical-Axon-on-Neuropixels-in-Kilosort) to directly examine in response to the short sparse noise stimulus the possible presence of TCA waveforms (22). This script allows to visualize waveforms with the highest rebounds as the most likely clusters being axons (*SI Appendix*, Extended Data Fig. 1*A*) and illustrates their locations on the probe (*SI Appendix*, Extended Data Fig. 1*C*, in red). Due to cortical mantle curvature, an extended layer 4 tangential insertion capturing large populations of TCAs and V1Ns is challenging to achieve, and this verification step is valuable to detect numerous axons. In case of low axon numbers, a reinsertion of the probe was attempted to achieve a higher yield. Identified axons usually have a rebound of sizes reaching −0.15 to −0.3 in the amplitude (a.u.) axis (*SI Appendix*, Extended Data Fig. 1*C* purple area). Second, an online mapping of RF confirmed the placement of the probe in the visually driven channels (32).

Altogether, the combination of RF mapping, indicating visually driven activity on a given set of channels, together with the Kilosort plotting, confirming putative TCA waveforms on the same visually driven channels, will both together give the confirmation that the tangential insertion is properly placed in layer 4 of the visual cortex, capturing visually driven TCA. For other sensory modalities, adapted stimuli should be employed to confirm the overlapping sensory responses to the location of the measured TCA.

### Spike Sorting, Axonal Detection, and Characterization.

Kilosort 2.5 was used for spike sorting. Manual curation was done post hoc using Phy2 (https://github.com/cortex-lab/phy/). During curation, clusters were not rejected based on multiple spatial peaks and too large spread. Furthermore, given the smaller amplitudes of TCA waveforms (Fig. 1*E*), the “MUA” labeling given by Kilosort was neglected in Phy2 when considering a putative TCA cluster. Quality controls were applied, including removal of double-counted spikes, interspike-interval violations (>0.05%), and isolation distance (isolation distance > 10 a.u., *SI Appendix*, Extended Data Fig. 3) (18). Quality metrics were obtained from ecephys (https://github.com/AllenInstitute/ecephys_spike_sorting). We evaluated the multichannel waveforms (MCW) by averaging the raw AP signal on all spike times from each individual single unit (up to 50,000 spikes), followed by a channel time offset correction of 2.78 μs between neighboring channels of the same analog–digital converter (13). Consequently, the MCW represents the spatiotemporal profile of the electrophysiological signal associated with the given single unit, over the entire tissue covered by the Neuropixels probe (Figs. 1*D* and 2A and *SI Appendix*, Extended Data Fig. 2*C*). As described previously (18), every cluster reaching all quality controls (Isolation distance, ISI violations, double-counted spikes, stability of firing in the awake recordings) was examined a custom-written Graphical User Interface (GUI) to label the obtained single-unit as either good V1N, good TCA, or an insufficient cluster. The decision to label a single unit as V1N or TCA was based on the presence of a larger rebound (a second peak) having a sufficient duration (>1 ms) and a sufficient spread (>10 channels), as reported in several figures (cf. Figs. 1*D*, 2*D*, and 4*A* and *SI Appendix*, Extended Data Figs. 1*A*, 6*D*, and 7). The careful observation of the MCW with all channels is therefore critical, where MCW should be plotted with an amplitude limit at 5 µV such that small amplitude will not be misclassified (cf. Fig. 1*E*).

For the pharmacological experiments (*n* = 5 mice) and the electrical experiments (*n* = 3 mice), a second custom-written GUI was used to distinguish clusters of different waveforms and different shapes due to either different clusters being wrongly merged between two different pharmacological conditions or electrical artifact waveforms changing the observed waveforms. Any clusters with noticeable different waveforms in different conditions were removed from further analysis. Once all controls were finished, the amplitude and the spread of each cluster were estimated. The signal-to-noise ratio (SNR) was calculated as the variability of the maximal amplitude in each channel. This SNR over all channels was then normalized to quantify the spread size as the number of channels above 0.1 (Fig. 1 *D* and *E*). When the probe was by chance aligned with the TCA axonal path running below cortical layer 6, the AP propagation speed can be estimated (*SI Appendix*, Extended Data Fig. 2) (18). Axonal propagation speed was measured using a minimal window of 5 channels away from the best channel and 5 channels away from the Neuropixels tip. Within this window, the AP peak was detected in a fixed time windows (−2.5 to −0.1 ms) just when its amplitude reached 4 SD of the measured baseline (−5 to −2.5 ms). A line was fitted between the different AP peak times in neighboring channels to determine the propagation speed; interpolation values above R = 0.725 were kept for quantification. This step was repeated on each of the four Neuropixels electrode columns independently; the best fit value was kept obtaining 7 distinguishable axonal paths showing an average of 0.92 ± 0.6 m/s propagation speed.

It is also possible to distinguish different firing properties of spiking between TCA and V1N by looking into their spiking variability. The variability of spiking was quantified by the different responses to a 1-s alternating 10° checker stimulus. The responses of TCA and V1N to 100 repeated trials (Fig. 1 *I*, *Top* and *Middle*) illustrate the higher trial-to-trial variability of V1N. This intertrial variability was divided by the average firing during the same period to estimate the Fano Factor of each V1N or TCA (23). Finally, to quantify the presence of local cortico-cortical axons in our dataset, we isolated monosynaptic cortico-cortical connections to unravel the presence of cortico-cortical axons which seem absent from our measurement (*SI Appendix*, Extended Data Fig. 5). To do so, monosynaptic pairs were first identified between V1N by way of CCG peak detection (cf. below). Then, the spikes that match the synaptic delay were isolated, and the waveform was recalculated on these very spikes in order to reveal timed-dependent coactivation in the observed waveforms. As expected, the postsynaptic firing appears in the waveform of the presynaptic neurons (*SI Appendix*, Extended Data Fig. 5 *D*, *E*, *G*, and *H*).

### Receptive Field and Functional Synaptic Connectivity.

To analyze visual responses properties, the RFs were first calculated from the peristimulus time histogram (PSTH) produced by summing the activity on each exposed pixel over the entire stimulation. Once this PSTH is produced, the RFs are the summation over time of the firing evoked on each pixel by the visual stimulus (10). RF contours were interpolated by a factor of two using the 2D-cubic-interpolation function from the SciPy package; only clusters with RFs showing a SNR (1/SD (RF) > 15) were kept for quantification (Fig. 4*B* and *SI Appendix*, Extended Data Fig. 3*A*).

Monosynaptic connections between TCA and V1N were detected using established methods (13). Cross-correlations were calculated using pycorrelate (https://github.com/tritemio/pycorrelate). To estimate the jitter, spike times over the entire recording were randomized within a consecutive 10 ms window and subtracted from the CCG. Reliable connections were detected from significant peaks within a window (0.5 to 4 ms for at least 4 consecutive time bins) above the baseline (3 SD, −4.5 to 0.5 ms, Fig. 2*C*). The distance on the probe between the TCA and the V1N was calculated between their respective peak channels (Fig. 5*A*). We then characterized diverging monosynaptic contact which were quantified over the populations as the number of functional contacts existing between one TCA and one to several V1N (Fig. 5*B*). Converging contact reflects the existence of one to several TCA monosynaptically connected to a single V1N (Fig. 5*C*). The synaptic efficacy of all monosynaptic contact was estimated from the jitter-corrected CCG as the area under the monosynaptic peak divided by the total number of spikes from the TCA (Fig. 5*D*) (18).

### Characterizing Thalamocortical and Cortico-cortical Axons in the AIlen Dataset.

To compare the thalamocortical and cortico-cortical axonal evoked signals, we downloaded the spiking data files from 56 sessions of the publicly available Allen Brain Institute Visual Coding dataset (https://allensdk.readthedocs.io/en/latest/visual_coding_neuropixels.html). From these recordings, we selected the units which are either in the dLGN or in one of the visual areas covered in the experiments (V1, LM, AL, RL, AM, and PM) measured across multiple Neuropixels probes. We then calculated the cross-correlation between all possible pairs across brain regions and identified putative long-range monosynaptic connections employing the method described above and using a 3 SD threshold. Since monosynaptic delays of long-range connections can reach several milliseconds in this dataset (27), the window for the peak detection was increased to 10 ms. Next, we selected a subset of experiments (*n* = 7, sessions: 719161530, 754829445, 755434585, 766640955, 778240327, 798911424, and 847657808) in which a number of long-range connections between different brain regions (thalamocortical or corticocortical) were found and downloaded the corresponding spike band datafiles (Allen Brain Observatory—Visual Coding AWS Public Data Set was accessed in June 2023 from https://registry.opendata.aws/allen-brain-observatory). Connections within the same brain region, e.g., within V1 were not studied in this dataset. Because the size of the raw spike band data of Neuropixels probe recordings is very large (~1TB in size per experimental session), we performed this analysis step only in a subset of sessions.

To estimate the signals evoked by long-range axons, we performed a spike-triggered analysis using the spike times of the presynaptic neuron onto the spike band data of the postsynaptic Neuropixels probe (Fig. 3 *B/C*, column 3). For quantifications, spikes times from the postsynaptic neuron should be removed from the presynaptic neuron spike times used to calculate the STA waveform (±1 ms) to avoid contamination from postsynaptic somatic spiking. This step forbids quantifying from axonal waveforms from local cortico-cortical pairs as both neurons are measured on the same probe in close proximity (*SI Appendix*, Extend data Fig. 5). From the spike band signal, we also calculated the multichannel spike waveforms of the presynaptic and postsynaptic neurons (Fig. 3 *B/C*, column one and three). Because not all axons evoked measurable signals, we used the channel of the postsynaptic neuron as the location on the postsynaptic Neuropixels probe to estimate the peak value of the presynaptic axon (Fig. 3*C*, horizontal dark gray dashed line). Furthermore, since the connection delay varied across pairs, we used the individual CCG peaks as a guide to determine the axonal response window (axonal response window = CCG peak time − 1 ms) (Fig. 3 *B/C*, vertical dark gray dashed line). The axonal response at this time point was used to calculate the peak value and the peak width (Fig. 3 *B/C*, axonal response across the 384 channels of the probe shown right of the colormap). For the thalamocortical axons, this analysis revealed a pronounced negative peak a few milliseconds after the presynaptic spike (Fig. 3 *D*, *Left*) and across neighboring channels (Fig. 3 *D*, *Right*) for which the amplitude and width could be estimated (Fig. 3*D*, green lines). The width was calculated as the number of channels that crossed the threshold of −075 μV in the STA (Fig. 3 *B*, *Right*, vertical green line). In case no channels passed this threshold, the peak width was assigned as 0 channels and is shown as not detected (n.d.) in Fig. 3*E*.

## Supplementary Material

Appendix 01 (PDF)

## Data Availability

The analysis used for the visualization of putative TCAs is available on the laboratory GitHub repository (34). Furthermore, the online analysis used to represent the visually driven MUA activity has been published in a separate publication (32). A preliminary recording has been made available online (35). Raw data have been deposited in Zenodo (10.5281/zenodo.3925903).

## References

[1] R. C. Reid, J.-M. Alonso, Specificity of monosynaptic connections from thalamus to visual cortex. Nature **378**, 281–284 (1995). 7477347 10.1038/378281a0

[2] J. Kremkow, J.-M. Alonso, Thalamocortical circuits and functional architecture. Annu. Rev. Vis. Sc. **4**, 1–23 (2018). 29856937 10.1146/annurev-vision-091517-034122 PMC7525828

[3] R. M. Bruno, B. Sakmann, Cortex is driven by weak but synchronously active thalamocortical synapses. Science **312**, 1622–1627 (2006). 16778049 10.1126/science.1124593

[4] C. M. Constantinople, R. M. Bruno, Deep cortical layers are activated directly by thalamus. Science **340**, 1591–1594 (2013). 23812718 10.1126/science.1236425 PMC4203320

[5] H. A. Swadlow, A. G. Gusev, Receptive-field construction in cortical inhibitory interneurons. Nat. Neurosci. **5**, 403–404 (2002). 11967546 10.1038/nn847

[6] Y. Bereshpolova, C. R. Stoelzel, C. Su, J.-M. Alonso, H. A. Swadlow, Activation of a visual cortical column by a directionally selective thalamocortical neuron. Cell Rep. **27**, 3733–3740.e3 (2019). 31242407 10.1016/j.celrep.2019.05.094 PMC7285384

[7] A. D. Lien, M. Scanziani, Cortical direction selectivity emerges at convergence of thalamic synapses. Nature **558**, 80–86 (2018). 29795349 10.1038/s41586-018-0148-5

[8] K. Reinhold, A. D. Lien, M. Scanziani, Distinct recurrent versus afferent dynamics in cortical visual processing. Nat. Neurosci. **18**, 1789–1797 (2015). 26502263 10.1038/nn.4153

[9] S. Najafian , A theory of cortical map formation in the visual brain. Nat. Commun. **13**, 2303 (2022). 35484133 10.1038/s41467-022-29433-y PMC9050665

[10] J. Kremkow, J. Jin, Y. Wang, J. M. Alonso, Principles underlying sensory map topography in primary visual cortex. Nature **533**, 52–57 (2016). 27120164 10.1038/nature17936 PMC4860131

[11] Y. J. Liew , Inferring thalamocortical monosynaptic connectivity in vivo. J. Neurophysiol. **125**, 2408–2431 (2021). 33978507 10.1152/jn.00591.2020 PMC8285656

[12] J.-S. Jouhanneau, J. Kremkow, A. L. Dorrn, J. F. A. Poulet, In vivo monosynaptic excitatory transmission between layer 2 cortical pyramidal neurons. Cell Rep. **13**, 2098–2106 (2015). 26670044 10.1016/j.celrep.2015.11.011 PMC4688033

[13] J.-S. Jouhanneau, J. Kremkow, J. F. A. Poulet, Single synaptic inputs drive high-precision action potentials in parvalbumin expressing GABA-ergic cortical neurons in vivo. Nat. Commun. **9**, 1540 (2018). 29670095 10.1038/s41467-018-03995-2 PMC5906477

[14] C. M. Niell, M. Scanziani, How cortical circuits implement cortical computations: Mouse visual cortex as a model. Annu. Rev. Neurosci. **44**, 1–30 (2021). 33914591 10.1146/annurev-neuro-102320-085825 PMC9925090

[15] M. M. Halassa, S. M. Sherman, Thalamocortical circuit motifs: A general framework. Neuron **103**, 762–770 (2019). 31487527 10.1016/j.neuron.2019.06.005 PMC6886702

[16] L. I. Schmitt , Thalamic amplification of cortical connectivity sustains attentional control. Nature **545**, 219–223 (2017). 28467827 10.1038/nature22073 PMC5570520

[17] J. J. Jun , Fully integrated silicon probes for high-density recording of neural activity. Nature **551**, 232–236 (2017). 29120427 10.1038/nature24636 PMC5955206

[18] J. Sibille , High-density electrode recordings reveal strong and specific connections between retinal ganglion cells and midbrain neurons. Nat. Commun. **13**, 5218 (2022). 36064789 10.1038/s41467-022-32775-2 PMC9445019

[19] H. A. Swadlow, A. G. Gusev, T. Bezdudnaya, Activation of a cortical column by a thalamocortical impulse. J. Neurosci. **22**, 7766–7773 (2002). 12196600 10.1523/JNEUROSCI.22-17-07766.2002 PMC6757983

[20] A. Antonini, M. Fagiolini, M. P. Stryker, Anatomical correlates of functional plasticity in mouse visual cortex. J. Neurosci. **19**, 4388–4406 (1999). 10341241 10.1523/JNEUROSCI.19-11-04388.1999 PMC2452998

[21] Y. Bereshpolova, X. Hei, J.-M. Alonso, H. A. Swadlow, Three rules govern thalamocortical connectivity of fast-spike inhibitory interneurons in the visual cortex. Elife **9**, e60102 (2020). 33289630 10.7554/eLife.60102 PMC7723404

[22] H. A. Swadlow, A. G. Gusev, The influence of single VB thalamocortical impulses on barrel columns of rabbit somatosensory cortex. J. Neurophysiol. **83**, 2802–2813 (2000). 10805678 10.1152/jn.2000.83.5.2802

[23] P. Kara, P. Reinagel, R. C. Reid, Low response variability in simultaneously recorded retinal, thalamic, and cortical neurons. Neuron **27**, 635–646 (2000). 11055444 10.1016/s0896-6273(00)00072-6

[24] D. J. Denman, D. Contreras, Complex effects on in vivo visual responses by specific projections from mouse cortical layer 6 to dorsal lateral geniculate. Nucleus. **35**, 9265–9280 (2015). 10.1523/JNEUROSCI.0027-15.2015 PMC4478248 26109652

[25] G. Born , Corticothalamic feedback sculpts visual spatial integration in mouse thalamus. Nat. Neurosci. **24**, 1711–1720 (2021). 34764474 10.1038/s41593-021-00943-0

[26] L. Wang, M. Kloc, E. Maher, A. Erişir, A. Maffei, Presynaptic GABAA receptors modulate thalamocortical inputs in layer 4 of rat V1. Cereb. Cortex **29**, 921–936 (2018), 10.1093/cercor/bhx364. PMC13209156 29373653

[27] J. H. Siegle , Survey of spiking in the mouse visual system reveals functional hierarchy. Nature **1**, 1–7 (2021). 10.1038/s41586-020-03171-x PMC10399640 33473216

[28] Y. K. Hong, I. Kim, J. R. Sanes, Stereotyped axonal arbors of retinal ganglion cell subsets in the mouse superior colliculus. J. Comp. Neurol. **519**, 1691–1711 (2011). 21452242 10.1002/cne.22595 PMC3652686

[29] T. McColgan , Dipolar extracellular potentials generated by axonal projections. Elife **6**, e26106 (2017), 10.7554/eLife.26106. 28871959 PMC5617635

[30] H. Peng , Morphological diversity of single neurons in molecularly defined cell types. Nature **598**, 174–181 (2021). 34616072 10.1038/s41586-021-03941-1 PMC8494643

[31] P. Shamash, M. Carandini, K. D. Harris, N. A. Steinmetz, A tool for analyzing electrode tracks from slice histology. Biorxiv [Preprint] (2018). 10.1101/447995 (Accessed 19 October 2018).

[32] J. Sibille, C. Gehr, K. L. Teh, J. Kremkow, Tangential high-density electrode insertions allow to simultaneously measure neuronal activity across an extended region of the visual field in mouse superior colliculus. J. Neurosci. Meth. **376**, 109622 (2022). 10.1016/j.jneumeth.2022.109622 35525463

[33] M. Pachitariu, N. Steinmetz, S. Kadir, M. Carandini, D. Harris Kenneth, Kilosort: Realtime spike-sorting for extracellular electrophysiology with hundreds of channels. Biorxiv [Preprint] (2016). 10.1101/061481 (Accessed 30 June 2016).

[34] KremkowLab, Axon on Neuropixels in Kilosort (GitHub, 2022).

[35] KremkowLab, KremkowLab/Axon-on-Neuropixels-in-Kilosort: Axon-on-Neuropixels-in-Kilosort (Version 1.0, Zenodo, 2022). 10.5281/zenodo.6839841.

