# Supplementary material for: Efficient mapping of the thalamocortical monosynaptic connectivity in vivo by tangential insertions of high-density electrodes in the cortex
Source: Proc Natl Acad Sci U S A. 2024 Jan 19;121(4):e2313048121. doi: 10.1073/pnas.2313048121 (PMC10823237; doi:10.1073/pnas.2313048121)
Supplement: Supplementary file 1 — Appendix 01 (PDF) [file pnas.2313048121.sapp.pdf]

Efficient mapping of the thalamocortical monosynaptic connectivity *in vivo* by tangential insertions of high-density electrodes in the cortex

(Supplementary Figures)

Jérémie Sibille[1-4#], Carolin Gehr[1-4], Jens Kremkow[1-4#]

[1]Neuroscience Research Center, Charité-Universitätsmedizin Berlin,10117 Berlin, Germany
[2]Bernstein Center for Computational Neuroscience Berlin, 10115 Berlin, Germany
[3]Institute for Theoretical Biology, Humboldt-Universität zu Berlin, 10115 Berlin, Germany
[4]Einstein Center for Neurosciences Berlin, 10117 Berlin, Germany
[#]Corresponding author: jeremie.sibille@charite.de; jens.kremkow@charite.de

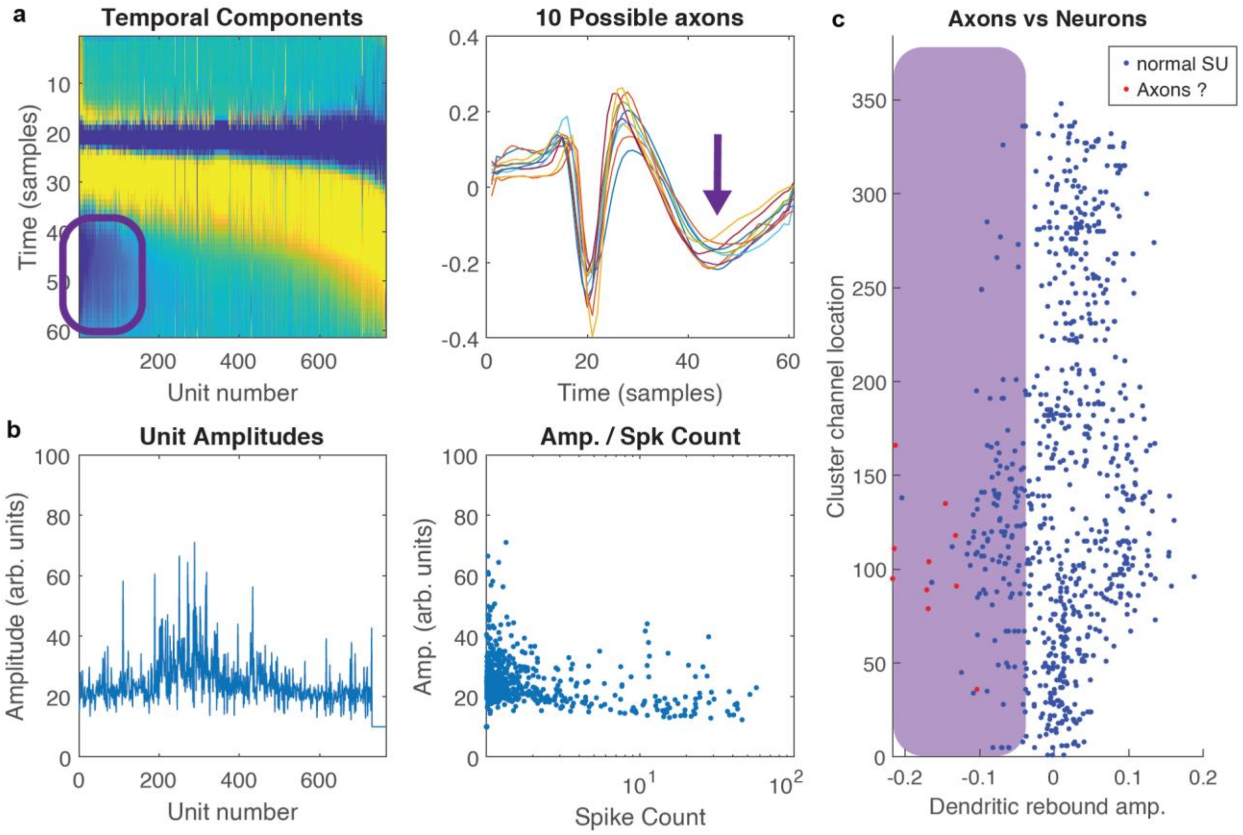

Extended Data Fig. 1: Adaptation of Kilosort to estimate the presence of TCA in a recording. a, Modified plots from the temporal components showing the captured waveforms, sorted by the amplitude of their rebound. Putative axons are located on the left with their negative rebound values highlighted in purple. Visualization of the first 10 waveforms with the highest rebound (right). **b**, Original Kilosort plots showing amplitude and spike count of the captured units. **c**, Each unit is shown with its channel location on the probe against their rebound amplitude in order to visualize the locations of putative axons (purple area). Note: the 10 clusters with highest rebound amplitude (from **a**) are highlighted in red. (https://github.com/KremkowLab/Cortical-Axon-on-Neuropixels-in-Kilosort)

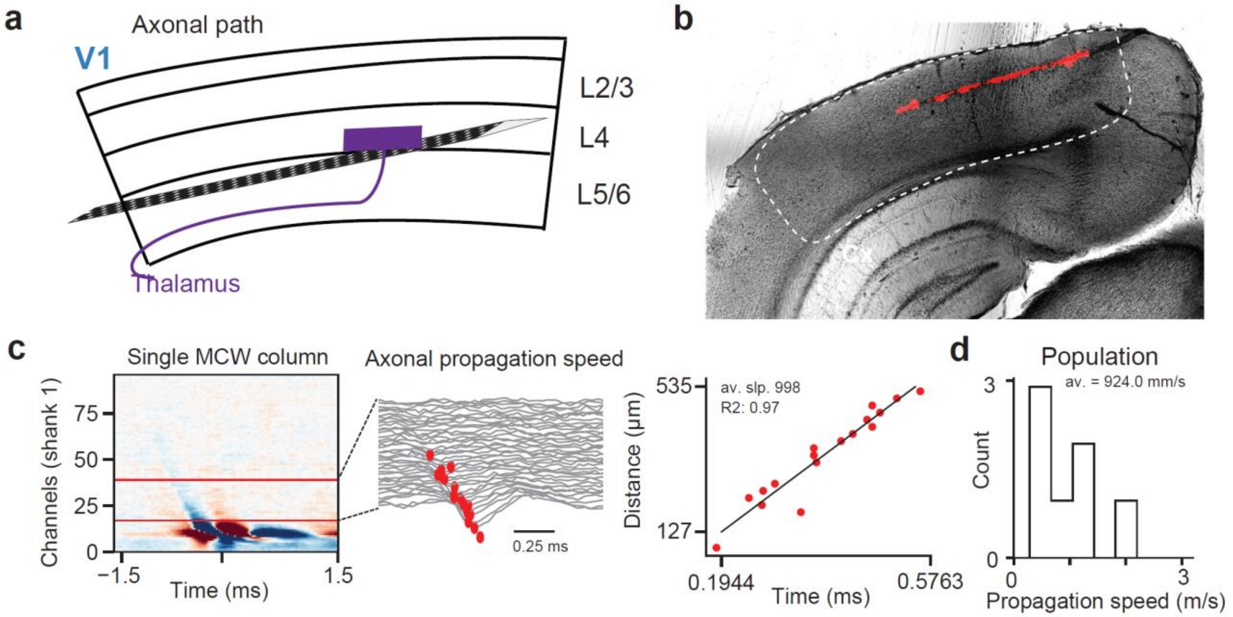

Extended Data Fig. 2: TCA propagation speed. a, Schematic of an insertion from the side. **b**, Coronal slice with Neuropixels probe track (in red). **c**, Quantification of the axonal propagation speed from the MCW (left). Zoom of the measured time points on which the axonal "tail" can be reliably detected (middle); together with the corresponding interpolation (right). **d**, Distribution of the conduction velocity (1 m/s, *n* = 7 TCAs, *n* = 3 mice).

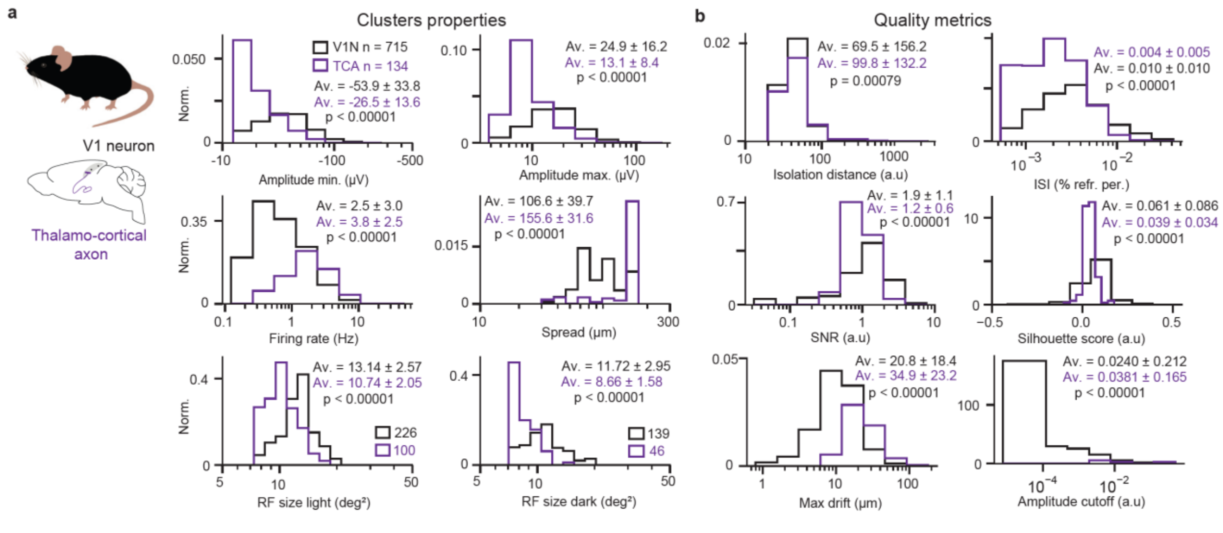

Extended Data Fig. 3: Properties and quality metrics of TCA & V1N. a, Cluster properties of V1N (black) and TCA (purple): Amplitudes are obtained from ecephys (negative - top left, positive - top right), both on log axis. Spread (middle right) and firing rate (middle left). Receptive field (RF) sizes were estimated from high SNR clusters (SNR > 15). **b**, Quality metrics for corresponding TCA and V1N.

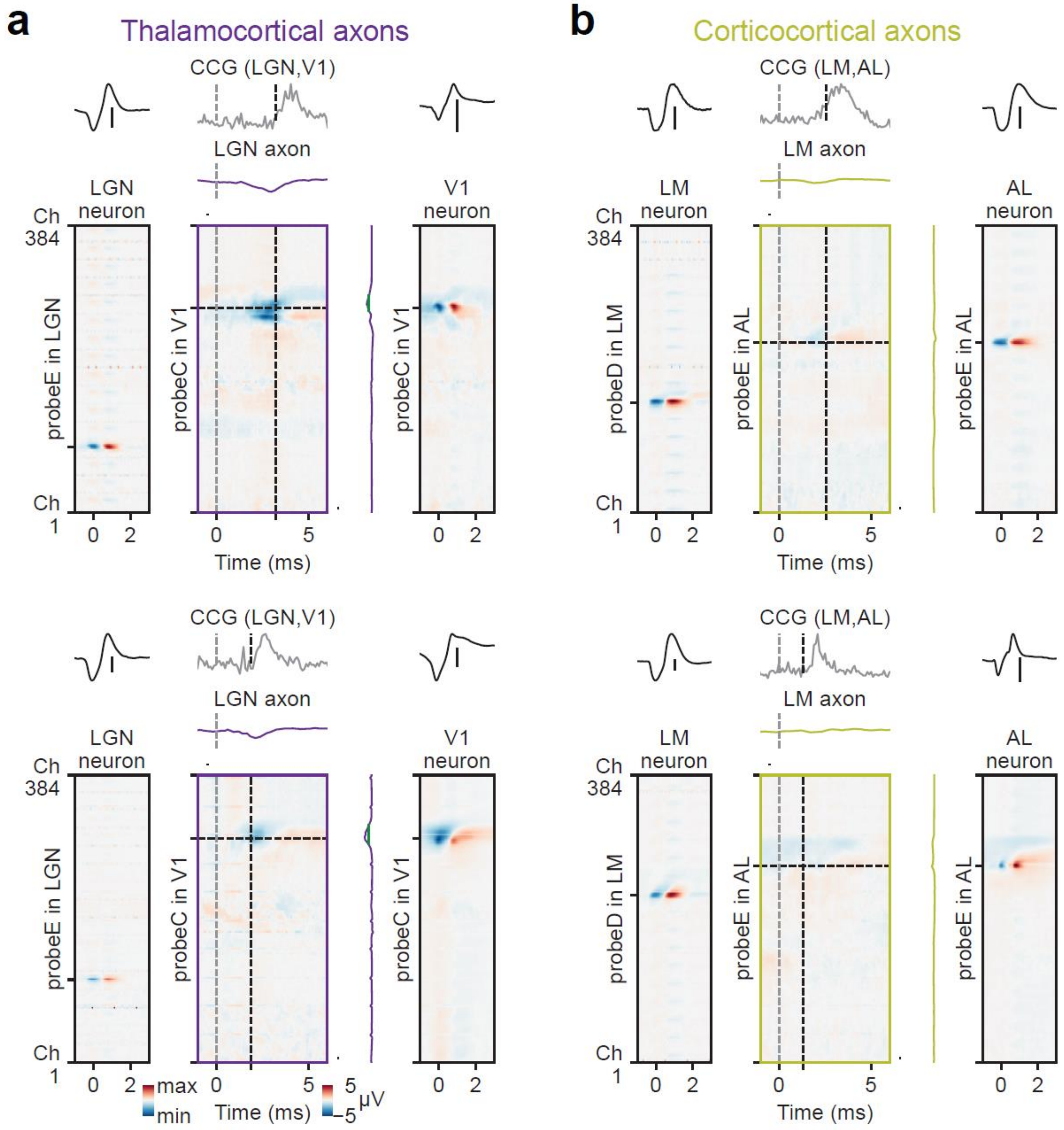

Extended Data Fig. 4: Examples of thalamocortical and long-range corticocortical axons. a, Examples of evoked STA waveforms in cortex by thalamocortical axons. The evoked negative waveform is visible at the recording sites of the postsynaptic neuron. **b**, Examples of signals evoked in cortex by corticocortical axons. The lack of axonal evoked signals is evident. a and b, same format as in Figure 3.

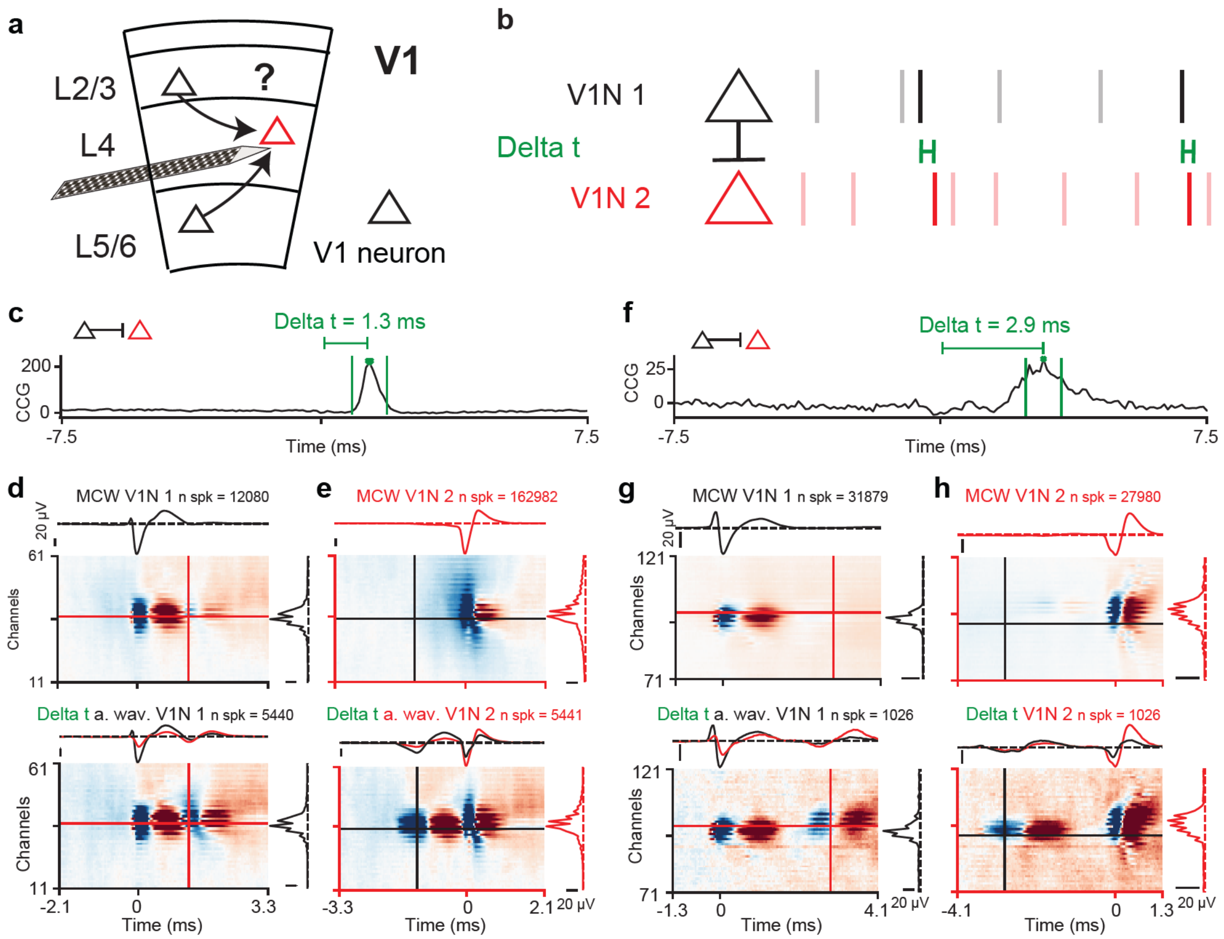

Extended Data Fig. 5: "Delta t averaged waveforms" from connected V1N pairs captured on a single probe. a, Schematic of the measured connectivity. **b**, Representation of two connected V1N firings (top V1N 1, black and below V1N 2, red) with the timing of the Delta t matching the synaptic delay observed in the CCG. Un-selected spikes that do not fall within the chosen Delta t delay are shaded. **c**, CCG between the two connected neurons with a synaptic delay of 1.3 ms. **d**, Multi channels waveform (MCW) for the presynaptic neurons (V1N 1, top). Averaged waveforms on Delta t delayed spikes from the pre-synaptic V1N 1 (bottom) reveals the waveform of the connected V1N 2 neuron. The amplitude profile of the pre-synaptic V1N 1 is represented in the top, and its minimal values on the left side. The position (best channel and time) of the post-synaptic neurons is annotated by red lines. **e**, MCW of V1N 2 (top) and the Delta t averaged waveform of the post-synaptic V1N 2 (bottom); similarly, the pre-synaptic waveform appears, without any in-between signals. **f-g-h**, Second example of another connected V1N pair, with longer delay (2.9 ms). In none of the studied cortico-cortical pairs (n = 267), any axonal waveform is visible, suggesting that corticocortical axons waveform is either of a different shape or below detection level. It is to be noticed, that axonal STA waveforms detection needs to remove the post-synaptic spike times from the spike times used to estimate the STA waveforms. This avoids contamination from post-synaptic somatic spikes but forbids here to calculate and STA waveforms as both spiking are occurring on the same probe.

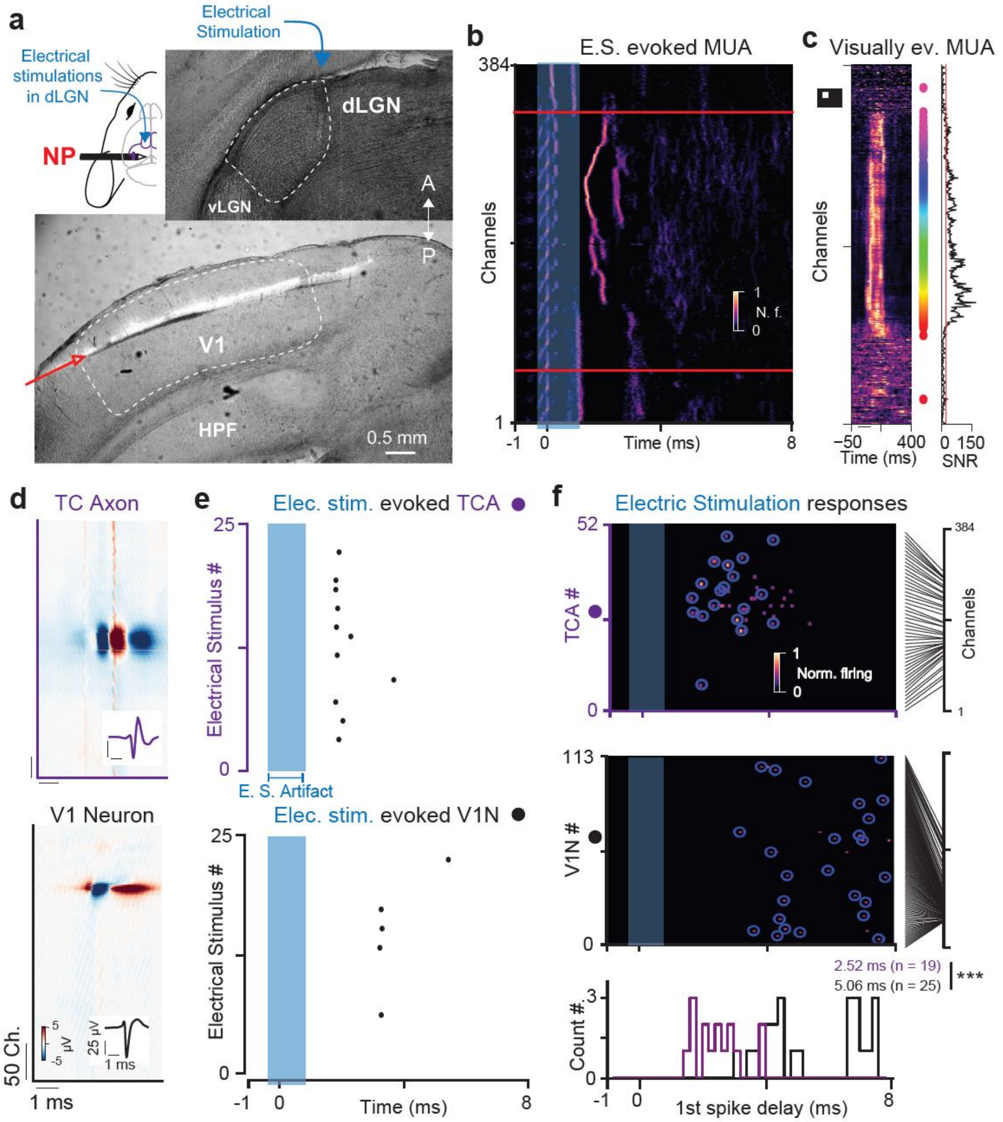

Extended Data Fig. 6: Electrical stimulations reveal a shorter response time of TCA waveforms strengthening their origin from dLGN neurons. a, Schematic of V1 tangential insertions from the side for electrical stimulations experiments (top left); Coronal slice showing the Neuropixels probe track (white, bottom) and the more anterior electrical-stimulation insertion (blue, no staining, top right). **b**, Representative MUA evoked by the electrical stimulations. Upon electrical stimulation an artifact will cover a window of -0.25 to + 1.0 ms indicated by the shaded blue area. The red lines indicate the electrically activated regions; clusters outside these regions are removed from further analysis. **c**, MUA evoked by sparse noise stimulus (left) and the corresponding visual response's SNR, indicating the visually driven channels (right). **d**, MCW of a TCA (top) and a V1N (bottom) with the corresponding waveform at the peak channel (lower inset). **e**, Raster plot of the TCA (top) and a V1N (middle) firing to 25 electrical stimulations; the electrical artifact is indicated by the shaded blue areas. **f**, Corresponding timing of the populations of TCA (top) and V1N (middle), illustrated with their respective channel position on the probe (right). Shown are their normalized firing responses over the 25 trials. The delays of the first spike of responsive TCA and V1N are indicated by a blue circle when a spike is evoked during this electrical stimulation protocol. The corresponding population first spike delay illustrates the shorter responses of TCA (2.52 ms) compared to V1N (5.06 ms). *** $p = 8.8\times10^{-13}$, n = 19 TCA, n = 25 V1N, n = 1 mouse. Two-sided Wilcoxon rank-sum test.

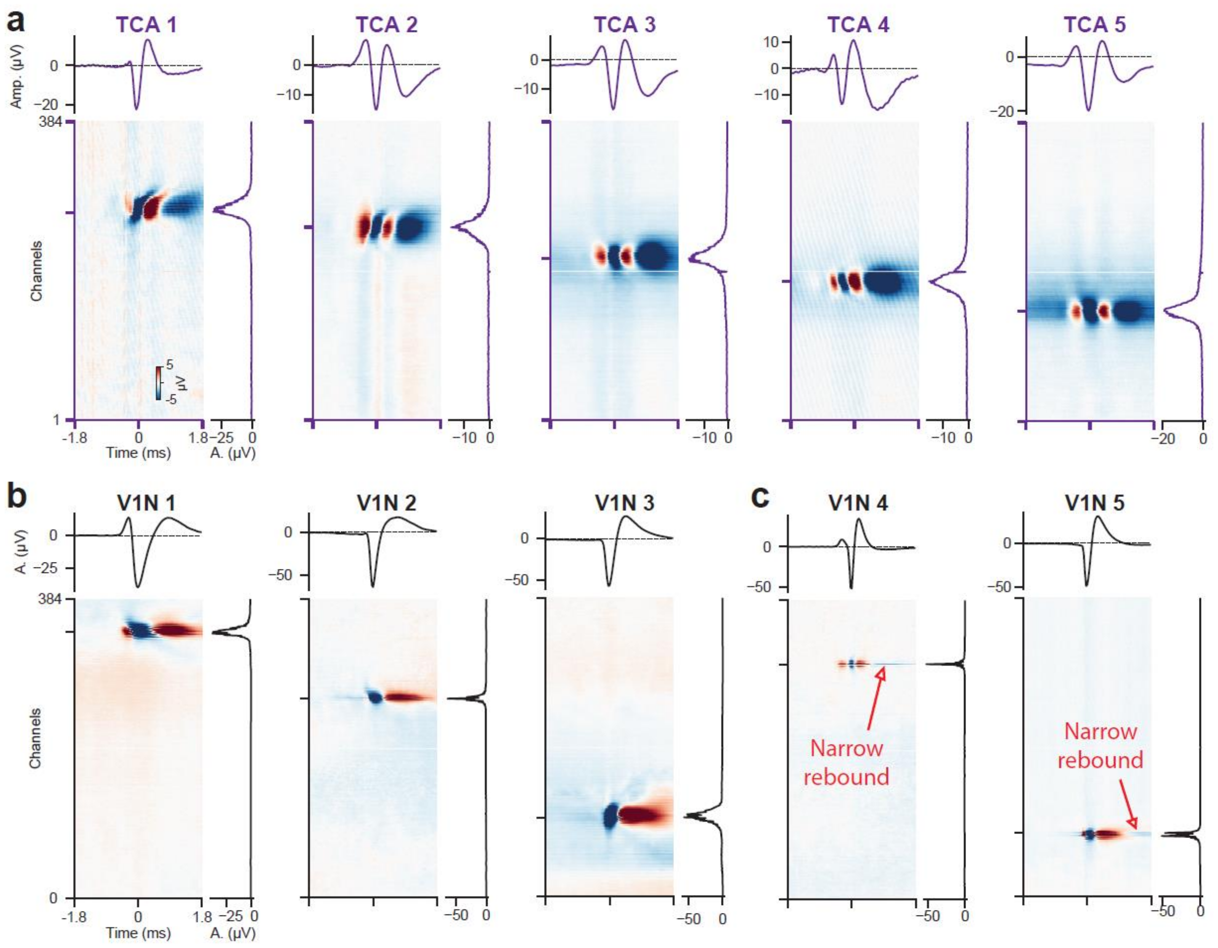

Extended Data Fig. 7: Examples of TCA and V1N. a, Detailed waveforms for 5 different TCA. The main color-coded plot is the Multi-channels waveform (MCW), illustrating the recalculated waveform on all channels of the probe when averaging the AP band on all spike times. The single channel waveform, chosen where the amplitude on the probe is the highest, is represented on top of the MCW; meanwhile its minimal amplitudes within each channels are shown on the right. **b**, Similar representation as in a, for 3 different V1Ns. **c**, Similar plotting for 2 V1Ns which are putative fast-spiking interneurons. Such waveforms sometimes can come with a negative "rebound" (red), however the small spread indicates it is a V1N and not a TCA.
